# Repeated bouts of pulmonary tuberculosis in a hunting cat: reinfection or recrudescence?

**DOI:** 10.1177/2055116921990292

**Published:** 2021-04-11

**Authors:** Carolina SC Albuquerque, Petra Černá, Danièlle A Gunn-Moore

**Affiliations:** 1 Royal (Dick) School of Veterinary Studies and The Roslin Institute, University of Edinburgh, Easter Bush Campus, Roslin, UK; 2 Department of Clinical Sciences, Colorado State University, Fort Collins, CO, USA

**Keywords:** Mycobacteria, *M microti*, pyogranulomatous, hypercalcaemia, tuberculosis, *Mycobacteriumtuberculosis* complex

## Abstract

**Case summary:**

A 7-year-old neutered male Siamese cat was referred for investigation of weight loss and hypercalcaemia (3.3 mmol/l; reference interval 2–3 mmol/l). Haematology, serum biochemistry, thoracic imaging, bronchoalveolar lavage (BAL), Ziehl–Neelsen staining of the BAL fluid and interferon gamma release assay (IGRA) were compatible with pneumonia caused by the less pathogenic member of the *Mycobacterium tuberculosis* complex, that is, *M microti* (the ‘vole bacillus’), which is common in cats in the UK. Treatment with azithromycin, rifampicin and marbofloxacin was given for 2 months, followed by 4 months of azithromycin and marbofloxacin. Treatment recommendations for tuberculous pneumonia have since changed. The cat remained asymptomatic for 1 year but went on to develop *M microti* pneumonia on five other occasions, and was treated for 6–12 months on each occasion. The patient’s clinical signs, hypercalcaemia and radiographic/CT pulmonary pathology always resolved completely, and the IGRA became negative, before antimycobacterial treatment was stopped. This suggests cure followed by reinfection owing to avid hunting behaviour. Alternatively, this could represent recrudescence of dormant disease. This case has previously been included in a study that described a series of cases of feline tuberculosis.

**Relevance and novel information:**

This case shows that *M microti* infection in cats can present as recurrent episodes of pneumonia, even after prolonged treatment courses.

## Case description

**Figure 1:**
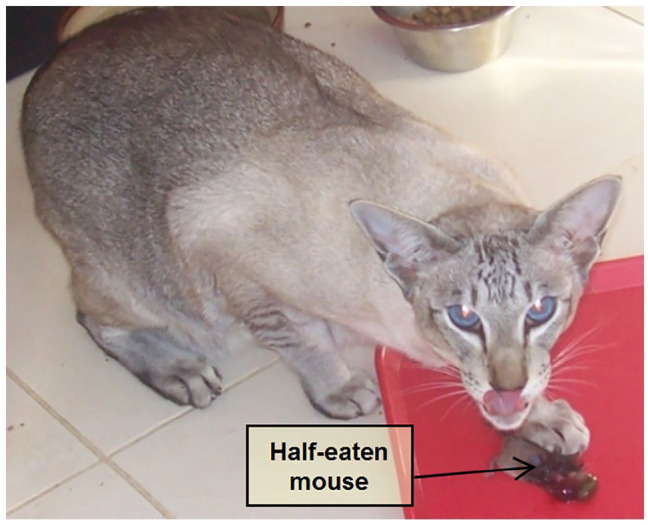
The 7-year-old neutered male Siamese cat, which has brought in a freshly hunted mouse to eat at its feeding station

A 7-year-old neutered male Siamese cat was presented to a university referral hospital in Scotland for weight loss and hyporexia of 1 month’s duration, as well as hypercalcaemia (3.3 mmol/l; reference interval [RI] 2–3 mmol/l) detected by the referring veterinarian. Defaecation, thirst and urination were normal. The cat was fed a good-quality commercial diet, and routine vaccinations and prevention against external and internal parasites were up to date. It was an indoor/outdoor cat and an avid hunter (Figure 1), with no travel history outside of Scotland. On physical examination, the cat had harsh lung sounds with a normal respiratory rate (25 breaths/min) and effort; the remainder of the physical examination, including a retinal examination, was unremarkable.

Differential diagnoses for hypercalcemia included granulomatous disease,^1,2^ neoplasia,^3,4^ hypervitaminosis D,^5^ renal disease,^3,6^ primary hyperparathyroidism,^3,7^ idiopathic hypercalcaemia,^8^ osteolysis^3^ or hypoadrenocorticism.^3,9^ Weight loss could be caused by hyporexia, maldigestion, malabsorption, chronic infection or inflammation, renal or hepatic disease, neoplasia, cardiac or – less likely – endocrine disease, including hyperthyroidism (the cat was relatively young for this), diabetes mellitus (polyuria, polydipsia and polyphagia would be expected) or hypoadrenocorticism (rare in cats);^10^ underfeeding, poor-quality diet and oral disease had been excluded. Harsh lung sounds could indicate pneumonia, primary or metastatic neoplasia or – less likely – idiopathic pulmonary fibrosis, pulmonary oedema or contusions. Hyporexia is a non-specific clinical sign; in the absence of oral/nasal disease or environmental stress, hyporexia could indicate systemic disease, nausea or pain.

Haematology, serum biochemistry (including thyroxine) and urine analysis were unremarkable, except for hypercalcaemia (ionised calcium [iCa] 1.75 mmol/l [RI 1.1–1.35 mmol/l]; Table 1). Ionised hypercalcaemia was confirmed with a repeated blood sample, and there was no haemolysis or lipolysis. Feline immunodeficiency virus antibody and feline leukaemia virus antigen were negative, and blood pressure was normal.

**Table 1:** Haematology and serum biochemistry results at presentation to the referral hospital

	Result	RI
WBCs (×10^9^/l)	7.3	7–20
Neutrophils (×10^9^/l)	5.1	2.5–12.8
Monocytes (×10^9^/l)	0.7	0.07–0.85
Eosinophils (×10^9^/l)	1.5	1.5–7
Basophils (×10^9^/l)	0	0–0.2
RBCs (×10^12^/l)	8.81	5.5–10
PCV (l/l)	0.45	0.24–0.45
Haemoglobin (g/dl)	13.9	8–14
MCV (fl)	51	39–55
MCHC (%)	30.9	30–36
Platelets (×10^9^/l)	310	300–600
Total protein (g/dl)	69	69–79
Albumin (g/dl)	32.2	28–39
Globulin (g/dl)	36.8	23–50
ALT (U/l)	56	15–60
ALP (U/l)	78	10–100
Bile acids (µmol/l)	3.5	0–7
Bilirubin (µmol/l)	3.3	0–6.8
Urea (mmol/l)	10.2*	2.8–9.8
Creatinine (µmol/l)	142	40–177
Total calcium (mmol/l)	3.4*	2.1–2.5
Ionised calcium (mmol/l)	1.75*	1.1–1.35
Phosphate (mmol/l)	2.1	1.4–2.5
Sodium (mmol/l)	150	145–156
Potassium (mmol/l)	4.6	4–5
Chloride (mmol/l)	114	109–122
Glucose (mmol/l)	6.3*	3.3–5

* Abnormal finding

RI = reference interval; WBCs = white blood cells; RBCs = red blood cells; PCV = packed cell volume; MCV = mean cell volume; MCHC = mean cell haemoglobin concentration; ALT = alanine transaminase; ALP = alkaline phosphatase

**Figure 2:**
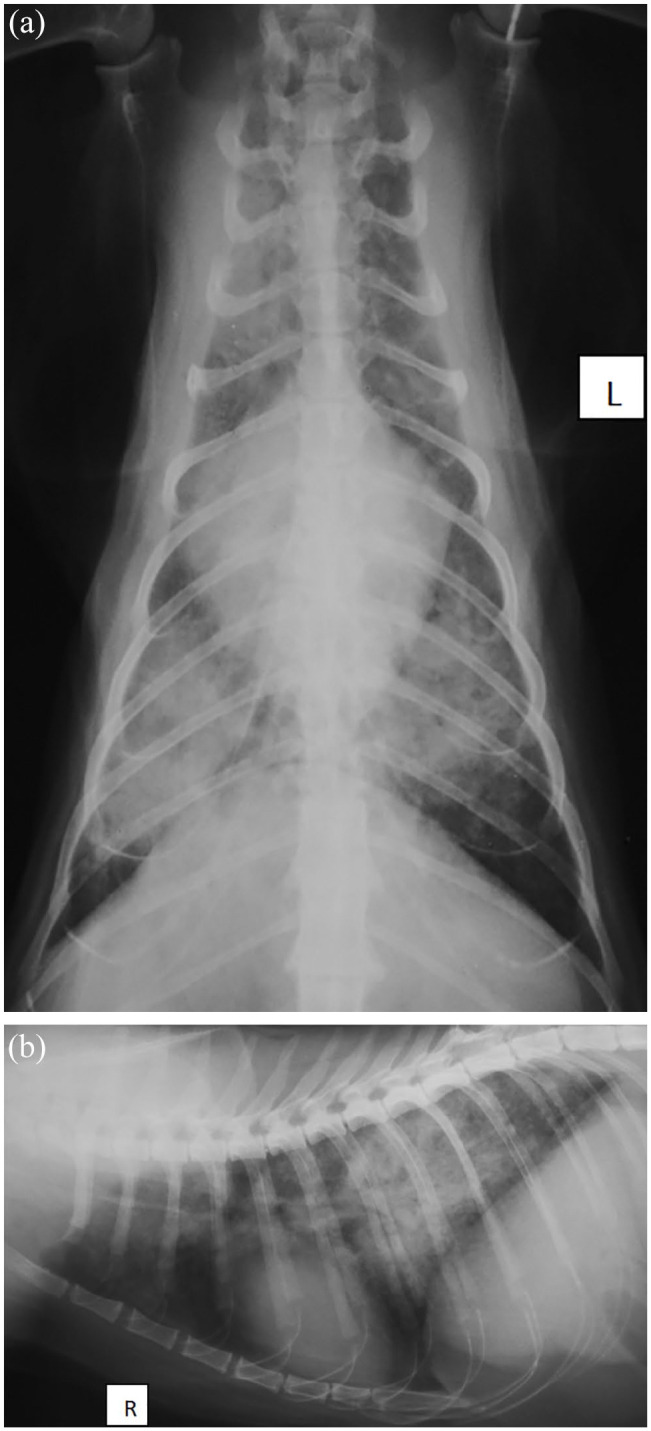
Dorsoventral (a) and right lateral thoracic (b) radiographs of the patient, showing a diffuse, interstitial–alveolar pattern, most marked on the caudal lung lobes

Further investigations of hypercalcaemia (Table 2) included plasma parathyroid hormone concentration (<10 pg/ml [RI <40 pg/ml]; not supporting hyperparathyroidism), plasma parathyroid hormone-related protein (<0.1 pmol/ml [RI <0.5 pmol/ml]; not supporting neoplasia, although there are other mechanisms by which neoplasia could result in hypercalcaemia), 25-hydroxyvitamin D (95 nmol/l [RI 127–335 nmol/l]; not supporting most types of hypervitaminosis D) and serum toxoplasma IgG and IgM titres (<50 and <20 [RI <50 and <20, respectively]). Abdominal ultrasound and radiographs were unremarkable. Thoracic radiographs (Figure 2) revealed a diffuse, interstitial–alveolar pattern, most marked on the caudal lung lobes. Differential diagnoses included infectious pneumonia (bacterial, parasitic, protozoal, viral or fungal), primary or metastatic neoplasia or, less likely, idiopathic pulmonary fibrosis. The spine and vertebrae were carefully examined in all radiographs for the presence of osteolytic lesions, and none were found.

**Table 2:** Plasma parathyroid hormone (PTH), parathyroid hormone-related protein (PTHrp), 25-hydroxyvitamin D and toxoplasma results

	Result	Reference interval
PTH (pg/ml)	<10	<40
PTHrp (pmol/ml)	<0.1	<0.5
25-hydroxyvitamin D (nmol/l)	95*	127–335
Toxoplasma IgG antibody titre by IFA	<50	<50
Toxoplasma IgM antibody titre by IFA	<20	<20

* Abnormal finding

IFA = immunofluorescence assay

**Figure 3:**
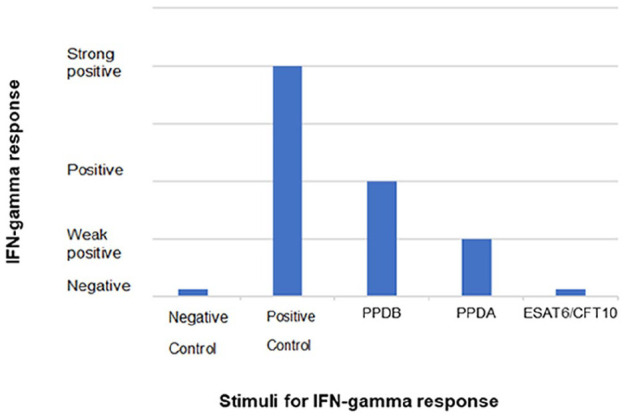
Interferon gamma release assay (IGRA) results. The response to bovine purified protein derivative (PPDB) indicates that the cat had a significant response to *Mycobacterium tuberculosis* complex (MTBC) bacteria; the response to avian purified protein derivative (PPDA) indicates that the cat had a weak immune response to members of the *Mycobacterium avium* complex (or this could be cross-reactivity with MTBC). There was no response, however, to ESAT6/CFT10 peptides, which are present in pathogenic MTBC bacteria, including *Mycobacterium bovis* and *M tuberculosis*. Hence, these IGRA results are compatible with infection with a less pathogenic member of the MTBC, most likely *Mycobacterium microti* in the UK. While *M bovis* infection can on occasion give this picture, the cat had never been outside of Scotland, which is officially *M bovis* free^11,12^

The patient received treatment for possible lungworms (Advocate; Bayer) and underwent bronchoscopy. The airways appeared macroscopically normal; bronchoalveolar lavage fluid (BALF) was sent for routine bacterial and fungal culture (which were negative), *Mycoplasma felis* PCR (this was negative) and cytology (which showed severe pyogranulomatous inflammation). In addition to routine haematoxylin and eosin staining, the BALF was stained with Grocott methenamine silver to evaluate the presence of fungi (negative) and Ziehl–Neelsen (ZN), which showed acid-fast bacilli morphologically consistent with mycobacterial infection. The interferon gamma release assay (IGRA) was performed, and the results were compatible with infection by the less pathogenic member of the *Mycobacterium tuberculosis* complex (MTBC); that is, *Mycobacterium microti* (‘the vole bacillus’) (Figure 3).

Combining clinical signs and results, the patient was diagnosed with pneumonia and hypercalcaemia caused by *M microti*; that is, the cat had a form of tuberculosis commonly seen in cats in certain UK regions, including Scotland.^13^

The patient was treated with rifampicin (Rifadin [Sanofi]; 10 mg/kg PO q24h), azithromycin (Zithromax [Pfizer]; 15 mg/kg PO q24h) and marbofloxacin (Marbocyl P [Vetoquinol]; 3 mg/kg PO q24h) for 2 months initially. A month after starting treatment, the cat’s body weight and appetite had improved, and iCa was normal. After 2 months of triple antibiotic therapy, haematology, serum biochemistry and thoracic radiographs were unremarkable, and rifampicin was stopped. After an additional 4 months, iCa and thoracic radiographs were unremarkable, IGRA was negative and serum vitamin D concentration was now normal, and so azithromycin and marbofloxacin were stopped.

**Figure 4:**
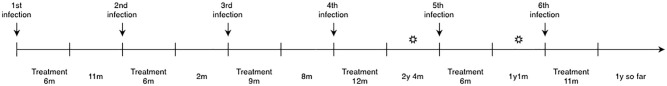
Timeline of the patient’s mycobacterial infections. The stars indicate when the presumptive *Mycoplasma felis* pneumonia episodes occurred. m = months; y = years

The patient remained asymptomatic for 1 year but was infected with tuberculous pneumonia five more times – a total of six episodes over one decade (Figure 4). The cat was tested for retroviruses on several occasions and the results were always negative. The longest the cat was asymptomatic while not receiving treatment between episodes of tuberculous pneumonia was 2 years 4 months. The cat always presented with weight loss, pneumonia, hypercalcaemia and an IGRA result compatible with *M microti*. In the initial infections, the cat was treated with triple antibiotic therapy (rifampicin, azithromycin and a fluoroquinolone – marbofloxacin or pradofloxacin) for a minimum of 2 months, then double therapy (azithromycin and a fluoroquinolone) for a minimum of 4 months. The last two episodes of tuberculous pneumonia were treated with triple antibiotic therapy for 6 and 11 months, including pradofloxacin (Veraflox [Bayer], 5 mg/kg PO q24h) and combined rifampicin/azithromycin capsules (Rifampicin 35 mg/Azithromycin 30 mg Capsules [Bova Laboratories]; rifampicin 12 mg/kg PO q24h and azithromycin 10 mg/kg PO q24h). All six episodes were treated for at least 2 months beyond clinical resolution. The cat was monitored throughout using an awake CT scan using the VetMouseTrap (University of Illinois). In addition to the six episodes of tuberculous pneumonia, the cat has had two episodes of presumptive *M felis* pneumonia (based on a negative IGRA and deep pharyngeal swab positive for *M felis* by PCR with a low cycle threshold number, hence significant infection; treated with pradofloxacin, dosed as above, for 2 months); at the time of writing, the cat has developed pulmonary fibrosis.

## Discussion

The case reported here has been previously included in a study that described a series of cases of feline tuberculosis;^14^ as the case is very unusual, we considered it important to describe it in detail and document how it evolved.

Mycobacterial disease in cats is perhaps underdiagnosed in veterinary medicine worldwide. Tuberculosis in cats, which is caused by either *M microti* or *M bovis*, is seen most commonly in hunting adult male cats and usually presents with cutaneous lesions (two-thirds of cases) and/or submandibular lymphadenopathy (half of cases), presumably due to bites while playing with prey or wound contamination.^13^ Other presentations include dyspnoea due to pneumonia, and gastrointestinal masses or abdominal lymphadenopathy, as reported recently in dozens of cats fed raw meat.^11^

Diagnosis of mycobacterial infection is often challenging. Apart from hypercalcaemia in some cases, the changes in haematology and serum biochemistry are non-specific, as they were in this case.^15^ Hypercalcaemia with granulomatous inflammation is due to local production of 1,25-hydroxyvitamin D by macrophages.^16^ Diagnostic imaging such as CT or radiography can be helpful in diagnosing mycobacterial infections in cats, although these can show a range of abnormalities and are not pathognomonic for mycobacterial infections.^16,17^ The most striking abnormality in this patient was a diffuse interstitial–alveolar pattern on thoracic radiographs, presumably resulting from haematogenous dissemination of mycobacteria.^17,18^ The ZN staining of the BALF confirmed mycobacterial disease in this case; however, it is important to note that a negative ZN stain is common in mycobacterial infection and does not rule it out.^13^ Where possible, identification of mycobacteria species should be attempted by a combination of PCR, culture and/or IGRA, as different species have different zoonotic risks, different prognosis and respond differently to antibiotic therapy.

Culture should be attempted from surgically excised or biopsied tissue. If the disease is only seen in the lungs, as seen in this case, a minimum of 5 ml of BALF is needed for mycobacteria culture. Mycobacteria are often slow-growing organisms, with certain species such as *M microti* requiring a minimum of 3 months to culture,^18^ and false-negative results are seen in up to half of feline cases.^13^

PCR on tissue or BALF is currently often used to diagnose mycobacterial infection, with a specificity of around 100% and variable sensitivity.^19^ However, this was not available at the time of the first diagnosis. It is important to keep frozen unfixed tissue from biopsied lesions where mycobacterial infection is a differential diagnosis, as neither culture nor PCR can be performed on formalin-fixed tissue.

The IGRA uses specific mycobacterial proteins to stimulate memory T cells in the blood ex vivo to determine if a patient has been infected with an organism that presents these peptides. The test has been validated in cats and its sensitivity to detect mycobacteria from the MTBC (including *M microti* and *M bovis*) is 80–100%^20,21^ and its specificity is 93% (J Mitchell and DA Gunn-Moore, unpublished data 2020).

Finally, molecular epidemiology tests such as spoligotyping, mycobacterial interspersed repetitive unit variable number tandem repeat or, ideally, whole-genome sequencing, would add enormously to the understanding of these cases. In particular, they would help to determine whether these cases are being continually re-infected from the environment (with different strains of mycobacteria), or whether it is more likely to be recrudescence (ie, the same strain); however, there could potentially be re-infection with the same strain, depending on the diversity of mycobacteria in the rodents the cats are hunting. For these molecular tests, extraction of DNA from cultured material is required. We did not have cultured material, which we acknowledge as a limitation in this case report. We were unable to obtain enough BALF in the first presentation, and the owners declined repeat BALs during subsequent episodes owing to the need for general anaesthesia and the possible risks.

This patient had a total of six episodes of tuberculous pneumonia over a decade; on each occasion there was resolution of clinical signs, hypercalcaemia, lung changes on imaging and the IGRA became negative before treatment was stopped. This is perhaps most suggestive of resolution of initial infection, followed by reinfection months to years later, due to avid hunting of infected mice and voles. Alternatively, this could represent recrudescence of dormant disease. In human medicine, the duration between apparent ‘cure’ and ‘relapse’ helps to assess whether disease is more likely to be reinfection vs recrudescence, with periods of less than 1 year between episodes being much more likely in recrudescent cases;^22^ it is not known whether this also applies to cats. A simple measure to prevent reinfection is to restrict the cats’ access to potential sources of reinfection, by keeping them indoors – this is the advice we give to all owners living in areas endemic for tuberculosis, but the owner of the cat reported here refused to do this.

It is possible that the cat’s negative IGRA (at the time of resolution of clinical signs) was a false-negative result; however, this result was always accompanied by resolution of the clinical signs, the hypercalcaemia and the imaging abnormalities. The negative predictive value of this test has been estimated to be 0.81 (95% confidence interval 0.64–0.91) (J Mitchell and DA Gunn-Moore, unpublished data 2020). More importantly, the IGRA remains positive in most cats treated for tuberculosis. This does not mean that those cats have been treated unsuccessfully, as they could be retaining primed memory T cells (J Mitchell and DA Gunn-Moore, unpublished data 2020). As in human medicine, the authors do not advise routine use of the IGRA for monitoring of disease resolution.

Of interest, the cat reported herein always showed the same presentation, with interstitial pneumonia being found on CT. This lung pattern is suggestive of haematogenous dissemination from a primary focus; however, no cutaneous lesion or peripheral lymphadenopathy were ever found in this case.

Previous recommendations for the treatment of tuberculosis in cats consisted of an initial phase of triple antibiotic therapy, followed by a continuation phase of double antibiotic therapy.^13^ There is limited evidence available on which to base optimal treatment; however, based on recommendations for people with tuberculosis, and experience gained treating feline mycobacterial infections for over 20 years, the authors now advise giving triple antibiotic therapy (rifampicin, azithromycin and pradofloxacin) for a minimum of 6 months when there is pulmonary involvement, and for 2 months beyond complete clinical resolution. The combined rifampicin and azithromycin capsule (Bova UK Laboratories) helps with compliance when medicating these cats. Side effects with rifampicin are common, and include hyporexia, vomiting, hepatopathy, hyperaesthesia, pruritus, erythema and skin discolouration or oedema;^23,24^ premediating with chlorphenamine, and giving concurrent S-adenosylmethionine or N-acetylcysteine may reduce the cutaneous irritation and hepatopathy.^25,26^ The main side effects of azithromycin are gastrointestinal and are uncommon.^24,27^ Long courses of pradofloxacin can result in diarrhoea, and neutropenia has been reported with high doses and prolonged courses of pradofloxacin in dogs.^28,29^ This patient was monitored for possible side effects by regular haematology and serum biochemistry, and given anti-nausea medication and hepato-protectants, as required.

## Conclusions

This case shows that tuberculous pneumonia due to *M microti* infection in cats can be seen as multiple bouts of reinfection, or recrudescence, even after long courses of treatment.
